# Criteria for selecting implementation science theories and frameworks: results from an international survey

**DOI:** 10.1186/s13012-017-0656-y

**Published:** 2017-10-30

**Authors:** Sarah A. Birken, Byron J. Powell, Christopher M. Shea, Emily R. Haines, M. Alexis Kirk, Jennifer Leeman, Catherine Rohweder, Laura Damschroder, Justin Presseau

**Affiliations:** 1 Department of Health Policy and Management, Gillings School of Global Public Health, The University of North Carolina at Chapel Hill, 1103E McGavran-Greenberg, 135 Dauer Drive, Campus Box 7411, Chapel Hill, NC 27599-7411 USA; 2 End-of-Life, Hospice, and Palliative Care Program, RTI International, 3040 Cornwallis Road, Research Triangle Park, NC 27709 USA; 3 School of Nursing, University of North Carolina at Chapel Hill, Chapel Hill, NC 27599 USA; 4 UNC Center for Health Promotion and Disease Prevention, The University of North Carolina at Chapel Hill, Chapel Hill, NC 27514 USA; 5 Ann Arbor VA Center for Clinical Management Research, Personalizing Options through Veteran Engagement (PROVE) QUERI Program, 2800 Plymouth Road, Building 16, Floor 3, Ann Arbor, MI 48109-2800 USA; 6 Clinical Epidemiology Program, Ottawa Hospital Research Institute, 501 Smyth Road, Ottawa, Ontario K1H 8L6 Canada; 7 School of Epidemiology and Public Health, University of Ottawa, 600 Peter Morand Crescent, Ottawa, Ontario K1G 5Z3 Canada; 8 School of Psychology, University of Ottawa, 136 Jean-Jacques Lussier – Vanier Hall, Ottawa, Ontario K1N 6N5 Canada

**Keywords:** Implementation theory, Theory, Framework, Criteria for selection

## Abstract

**Background:**

Theories provide a synthesizing architecture for implementation science. The underuse, superficial use, and misuse of theories pose a substantial scientific challenge for implementation science and may relate to challenges in selecting from the many theories in the field. Implementation scientists may benefit from guidance for selecting a theory for a specific study or project. Understanding how implementation scientists select theories will help inform efforts to develop such guidance. Our objective was to identify which theories implementation scientists use, how they use theories, and the criteria used to select theories.

**Methods:**

We identified initial lists of uses and criteria for selecting implementation theories based on seminal articles and an iterative consensus process. We incorporated these lists into a self-administered survey for completion by self-identified implementation scientists. We recruited potential respondents at the 8th Annual Conference on the Science of Dissemination and Implementation in Health and via several international email lists. We used frequencies and percentages to report results.

**Results:**

Two hundred twenty-three implementation scientists from 12 countries responded to the survey. They reported using more than 100 different theories spanning several disciplines. Respondents reported using theories primarily to identify implementation determinants, inform data collection, enhance conceptual clarity, and guide implementation planning. Of the 19 criteria presented in the survey, the criteria used by the most respondents to select theory included analytic level (58%), logical consistency/plausibility (56%), empirical support (53%), and description of a change process (54%). The criteria used by the fewest respondents included fecundity (10%), uniqueness (12%), and falsifiability (15%).

**Conclusions:**

Implementation scientists use a large number of criteria to select theories, but there is little consensus on which are most important. Our results suggest that the selection of implementation theories is often haphazard or driven by convenience or prior exposure. Variation in approaches to selecting theory warn against prescriptive guidance for theory selection. Instead, implementation scientists may benefit from considering the criteria that we propose in this paper and using them to justify their theory selection. Future research should seek to refine the criteria for theory selection to promote more consistent and appropriate use of theory in implementation science.

## Background

Theories and frameworks offer an efficient way of generalizing findings across diverse settings within implementation science [1]. Theories and frameworks (see Department of Veterans Health Administration’s Quality Enhancement Research Initiative [2013] for a taxonomy of theories, frameworks, and models, hereafter “theories”) generalize findings by providing a synthesizing architecture—that is, an explicit summary of explanations of implementation-related phenomena to promote progress and facilitate shared understanding [2]. Furthermore, theories guide implementation, facilitate the identification of determinants of implementation, guide the selection of implementation strategies, and inform all phases of research by helping to frame study questions and motivate hypotheses, anchor background literature, clarify constructs to be measured, depict relationships to be tested, and contextualize results [3].

Given their potential benefits, the underuse, superficial use, and misuse of theories represent a substantial scientific challenge for implementation science [4–8]. In one review, Tinkle et al. [4] highlighted pervasive *underuse of theory* (i.e., not using a theory at all); most of the large National Institutes of Health-funded projects that they reviewed did not use a theory. Likewise, a review of evaluations of guideline dissemination and implementation strategies from 1966 to 1998 showed that only a minority (23%) used any theory [5]. A scoping review of guideline dissemination strategies to physicians covering 2006 to 2016 showed that, although theory use had increased over time, fewer than half (47%) of included studies used a theory [8]. While theory use appears to be on the rise, it remains underused. Kirk et al. [9] reviewed studies citing the Consolidated Framework for Implementation Research and found that few applied the framework in a meaningful way (i.e., *superficial use*). For example, many articles cited the Consolidated Framework for Implementation Research (CFIR) in the “Background” or “Discussion” sections to acknowledge the complexity of implementation but did not apply the CFIR to data collection, analysis, or reporting findings. Similar results were found for studies conducted through 2009 that cited the use of the Promoting Action on Research Implementation in Health Services (PARIHS) framework [10]. Gaglio et al. [11] found that the most frequently studied dimension of the Reach Effectiveness Adoption Implementation Maintenance (RE-AIM) framework (reach) was often used incorrectly (i.e., *misuse*) [12]. Reach compares intervention participants (numerator) to non-participants (denominator). Examples of misuse include comparisons of participants to each other rather than to non-participants (e.g., [13]). The underuse, superficial use, and misuse of implementation theories may limit both the field’s advancement and its capacity for changing healthcare practice and outcomes.

The underuse, superficial use, and misuse of theories may relate, in part, to the challenge of selecting from among the many that exist in the field [14, 15], each with its own language and syntax, and varying levels of operationalized definitions [16] and validity [17]. Implementation researchers and practitioners (i.e., implementation scientists) have at their disposal myriad theories developed within traditional disciplines (e.g., sociology, health services research, psychology, management science) and increasingly within implementation science itself [18]. A move toward synthesizing theories may address the overlap; however, the question of which theory to select remains [19]. Therefore, implementation scientists would benefit from guidance for selecting a theory for a specific project. Guidance for theory selection may encourage implementation scientists to use theories, discouraging underuse; to use theories meaningfully, discouraging superficial use; and to be mindful of the strengths, weaknesses, and appropriateness of the theories that they select, discouraging misuse. Guidance for theory selection will promote theory testing and identification of needs around theory development, contributing to the advancement of the science. Indeed, applying theory meaningfully provides an opportunity to test, report, and enhance its utility and validity and provides evidence to support adaptation or replacement. As a first step toward the development of guidance for theory selection, this study aimed to identify which theories implementation scientists use, how they use theories, and the criteria used to select theories.

## Methods

### Survey design, instrument, and procedure

We conducted an observational study of implementation scientists using a self-administered paper and web-based survey. To create the survey instrument, we identified potential uses and criteria for selecting implementation theories using seminal texts and an iterative review process. SB, BP, and JP began with two seminal articles [20, 21] and one conference presentation [22]. Building on these texts, SB, BP, JP, and CS iteratively refined the uses and criteria for selecting theory through independent review in which authors identified uses and criteria for selecting theory, clarified definitions, and eliminated redundancies and then reconciled disagreements through an informal consensus process. The final 19-item instrument incorporated the resulting 12 potential uses (Table 1) and 19 criteria for selecting implementation theories (Table 2). In the survey, we asked respondents to identify:

1. Their demographic and professional characteristics.
2. Theories that they have used as part of their implementation research or practice (open-ended).
3. The ways in which they have used theories (e.g., to inform data collection, analysis) (select all relevant options).
4. Criteria that they use to select a theory (e.g., analytic level, disciplinary origins) (select all relevant options provided based on the seminal text and iterative review and consensus process described above). In addition to the options provided, we included open-ended items to identify criteria that respondents used to select theories other than those listed in the survey. We also asked respondents to rank the top three criteria that they use to select a theory.
5. Any additional thoughts that they had regarding theory selection not addressed in other survey items (open-ended).

**Table 1:** Ways in which theories have been used (*n* = 223)

Ways in which theories have been used	Percent
1. To identify key constructs that may serve as barriers and facilitators	80.09
2. To inform data collection	77.06
3. To guide implementation planning	66.23
4. To enhance conceptual clarity	66.23
5. To specify the process of implementation	63.20
6. To frame an evaluation	61.04
7. To inform data analysis	59.74
8. To guide the selection of implementation strategies	58.87
9. To specify outcomes	55.84
10. To clarify terminology	48.05
11. To convey the larger context of the study	48.05
12. To specify hypothesized relationships between constructs	47.62
None of the above	0.00

**Table 2:** Criteria used for selecting theory (*n* = 212)

Criterion and definition	Percent
1. Analytic level, e.g., individual, organizational, system	58.02
2. Logical consistency/plausibility, i.e., inclusion of meaningful, face-valid explanations of proposed relationships	56.13
3. Description of a change process, i.e., provides an explanation of how changes in process factors lead to changes in implementation-related outcomes	53.77
4. Empirical support, i.e., use in empirical studies with results relevant to the framework or theory, contributing to cumulative theory-building	52.83
5. Generalizability, i.e., applicability to various disciplines, settings, and populations	47.17
6. Application to a specific setting (e.g., hospitals, schools) or population (e.g., cancer)	44.34
7. Inclusion of change strategies/techniques, i.e., provision of specific method(s) for promoting change in implementation-related processes and/or outcomes	44.34
8. Outcome of interest, i.e., conceptual centrality of the variable to which included constructs are thought to be related	41.04
9. Inclusion of a diagrammatic representation, i.e., elaboration in a clear and useful figure representing the concepts within and their interrelations	41.04
10. Associated research method (e.g., informs qualitative interviews, associated with a valid questionnaire or methodology for constructing one), i.e., recommended or implied method to be used in an empirical study that uses the framework or theory	40.09
11. Process guidance, i.e., provision of a step-by-step approach for application	38.68
12. Disciplinary approval, i.e., frequency of use, popularity, acceptability, and perceptions of influence among a given group of scholars or reviewers, country, funding agencies, etc.; endorsement or recommendation by credible authorities in the field	33.96
13. Explanatory power/testability, i.e., ability to provide explanations around variables and effects; generates hypotheses that can be empirically tested	32.55
14. Simplicity/parsimony, i.e., relatively few assumptions are used to explain effects	32.08
15. Specificity of causal relationships among constructs, i.e., summary, explanation, organization, and description of relationships among constructs	32.08
16. Disciplinary origins, i.e., philosophical foundations	18.40
17. Falsifiability, i.e., verifiable; ability to be supported with empirical data	15.09
18. Uniqueness, i.e., ability to be distinguished from other theories or frameworks	12.74
19. Fecundity, i.e., offers a rich source for generating hypotheses	9.91
None of the above	0.00

We recruited potential respondents at the 8th Annual Conference on the Science of Dissemination and Implementation in Health (2015) in Washington DC, USA, and via several international email lists (Table 3). We contacted potential respondents beginning in December 2015 and closed the survey at the end of February 2016. Potential respondents recruited at the 8th Annual Conference on the Science of Dissemination and Implementation in Health (2015) had the opportunity to complete a paper survey or to receive a link to a web-based version of the survey; all other potential respondents had access to the web-based version of the survey (Qualtrics, Provo, UT). We randomized response options in the web-based version of the survey to minimize bias associated with standard item ordering.

**Table 3:** Email lists

Organization	Approximate readership
Alberta SPOR (Strategy for Patient Oriented Research) KT Platform newsletter	250
Association of Behavioral and Cognitive Therapies Dissemination and Implementation Science Special Interest Group	269
Australasian Implementation Conference listserv	Unknown
Editorial board of *Implementation Science*	77
European Implementation Collaborative	300
Self-identified implementation researchers in the University of North Carolina’s School of Public Health	15
Implementation Network	2400
Implementation Research Institute fellows and faculty	51
Knowledge Utilization Studies Program FYI newsletter	150
Mentored Training in Dissemination and Implementation Research in Cancer (MT-DIRC) alumni and faculty	39
Nordic Implementation Network	200
Society for Implementation Research Collaboration (SIRC) Network of Expertise	107
Triangle Implementation Science listserv	123
University of North Carolina at Chapel Hill Implementation Science student listserv	77

### Ethics, consent, and permissions

Before completing the survey, participants read information regarding the study, including their right not to complete the survey and that doing so implied their consent. The institutional review board at the University of North Carolina at Chapel Hill exempted the study from human subject review.

### Analysis

We conducted a descriptive analysis to describe which theories respondents used, the ways in which they used theories, and criteria that they considered when selecting theories. Analyses were conducted using Stata/IC statistical software v14.1. We used inductive content analysis to identify criteria that respondents used to select theories other than those listed in the survey and analyze respondents’ thoughts regarding theory selection not addressed in other survey items [23]. EH conducted the initial analysis, and SB, BP, JP, and CS collaborated on identifying criteria not represented elsewhere in the survey and salient quotes regarding theory selection.

## Results

### Respondent characteristics

After deleting observations for which the majority of survey items were incomplete, the study sample consisted of 223 survey respondents. Demographic characteristics of survey respondents are displayed in Table 4. Respondents included implementation researchers (42%), implementation practitioners (11%), and those who identified as both implementation researchers and practitioners (48%). The majority was female (72%) and white (90%). Other races represented include Asian (5%), black or African American (1%), and “other” or multiple races (4%). Survey respondents represented 12 different countries, with slightly more than half of respondents (55%) reporting the USA as the country in which their institution was located. Other commonly reported countries included Australia (18%), Canada (9%), the UK (7%), and Sweden (5%). Most respondents (67%) reported a PhD as their highest degree earned; 21% reported a Master’s degree; other respondents reported MD, Bachelor’s degree, and “Other.”

**Table 4:** Respondent characteristics

Respondent characteristics	Percent
Research/practice (*n* = 223)	
Research	41.70
Practice	10.76
Both	47.53
Sex (*n* = 186)	
Female	71.51
Male	10.05
Other	0.54
Race (*n* = 180)	
White/Caucasian	90.00
Black/African American	0.56
Asian	5.00
Other/multiple	4.44
Ethnicity (*n* = 181)	
Non-Hispanic	98.90
Hispanic	1.10
Institution country (*n* = 181)	
USA	54.70
Australia	17.68
Canada	9.39
UK	7.18
Sweden	4.97
Denmark	1.66
Ireland	1.10
Netherlands	0.55
Nepal	0.55
Austria	0.55
Highest degree obtained (*n* = 186)	
PhD	67.20
Master’s	20.97
MD	5.91
Bachelor’s	3.23
Other	2.69
Institution type (*n* = 182)	
Academic	72.53
Hospital-based research institute	14.29
Government	13.74
Service provider	13.74
Other	8.24
Industry	2.75
Seniority (*n* = 182)	
Years conducting research [mean (SD)]	13.8 (8.9)
Years conducting implementation research [mean (SD)]	7.4 (7.1)
Published papers [mean (SD)]	36.6 (61.4)
Published papers in implementation [mean (SD)]	10.2 (18.7)
Has been principal investigator of externally funded research study	63.74
Training discipline	
Mental health/social work	71.43
Public health/policy	51.02
Arts and sciences	33.67
Healthcare	28.57
Education	5.10
Work discipline	
Public health/policy	79.59
Mental health/social work	35.71
Healthcare	19.39
Other	9.18
Education	4.08

*SD* standard deviation

Most survey respondents (73%) were based at academic institutions. Other institution types included hospital-based research institutes (14%), government (14%), service providers (e.g., hospitals and public health agencies) (14%), and industry (e.g., contract research organizations) (3%). Respondents reported spending a mean = 14, standard deviation (SD) = 8.9 years conducting research and a mean = 7, SD = 7.1 years conducting implementation research, specifically. They reported having published a mean = 37, SD = 61.4 papers overall and a mean = 10, SD = 18.7 papers related to implementation science. Most respondents (64%) reported having been principal investigator of an externally funded research study.

### Theories used

Survey respondents reported using more than 100 different theories from several disciplines including implementation science, health behavior, organizational studies, sociology, and business. The most commonly listed included the Consolidated Framework for Implementation Research (CFIR), Theoretical Domains Frameworks (TDF), PARIHS, Diffusion of Innovations, RE-AIM, Quality Implementation Framework, and Interactive Systems Framework (Table 5). Additionally, many respondents reported using theories in combination and theories developed “in-house.”

**Table 5:** Theories used

Theory	Percent
Consolidated Framework for Implementation Research	20.63
Reach Effectiveness Adoption Implementation Maintenance	13.90
Diffusion of Innovation	8.97
Theoretical Domains Framework	5.38
Exploration, Preparation, Implementation, Sustainment	4.93
Proctor’s Implementation Outcomes	4.93
Organizational Theory of Implementation of Innovations	3.59
Knowledge to Action	3.14
Implementation Drivers Framework	3.14
Active Implementation Framework	2.69
Theory of Planned Behaviour	2.69
Behaviour Change Wheel	2.69
Normalization Process Model	2.69
PARIHS	1.79
Social Cognitive Theory	1.79
Intervention Mapping	1.79
Interactive Systems Framework	1.79
Organizational Readiness Theory	1.79
Replicating Effective Programs	1.35
Social Ecological Framework	1.35
QUERI	1.35
PBIS	1.35
Social Learning Theory	1.35
Other	4.04

### Ways in which theories are used

The most common ways in which survey respondents used theories in their implementation work were to identify key constructs that may serve as barriers and facilitators (80%), to inform data collection (77%), to enhance conceptual clarity (66%), and to guide implementation planning (66%). Respondents also used theories to inform data analysis, to specify hypothesized relationships between constructs, to clarify terminology, to frame an evaluation, to specify implementation processes and/or outcomes, to convey the larger context of the study, and to guide the selection of implementation strategies (Table 1).

### Criteria that implementation scientists use to select theories

On average, survey respondents reported having used 7 (mean = 7.04; median = 7) of the 19 criteria listed in the survey when selecting an implementation theory. Some reported having used all 19 (Table 2). The criteria used by the most respondents included *analytic level* (58%), *logical consistency/plausibility* (56%), *empirical support* (53%), and *description of a change process* (54%). The criteria used by the fewest respondents included *fecundity* (10%), *uniqueness* (12%), and *falsifiability* (15%).

#### Criteria ranking

We eliminated responses with fewer than two criteria ranked (*n* = 48). Thirty-three percent of respondents ranked *empirical support* as one of the three most important criteria, but only 17% ranked it number 1 (see Table 6). *Application to a specific setting/population and explanatory* and *power/testability* were ranked second and third, respectively.

**Table 6:** Criteria ranking (*n* = 175)

Criterion	First most important (%)	Second most important (%)	Third most important (%)	Total (%)
Empirical support	16.57	11.43	5.14	33.14
Application to a specific setting/population	13.71	8.00	4.57	26.29
Explanatory power/testability	12.57	5.71	6.29	24.57
Description of a change process	10.86	9.14	4.57	24.57
Analytic level	8.00	11.43	7.43	26.86
Specificity of a causal relationship among constructs	6.86	5.71	6.29	18.86
Logical consistency/plausibility	6.29	5.71	5.71	17.71
Generalizability	5.14	5.14	9.71	20.00
Process guidance	5.14	7.43	10.86	23.43
Outcome of interest	4.00	3.43	4.57	12.00
Other criteria	4.00	3.43	4.00	11.43
Disciplinary approval	2.86	4.57	3.43	10.86
Associated research method	1.14	6.29	6.29	13.71
Simplicity/parsimony	1.14	4.00	5.14	10.29
Disciplinary origins	0.57	1.14	2.29	4.00
Falsifiability	0.57	2.86	1.14	4.57
Inclusion of change strategies/techniques	0.57	2.86	4.00	7.43
Fecundity	0.00	1.71	0.57	2.29
Inclusion of a diagrammatic representation	0.00	0.00	4.57	4.57
Uniqueness	0.00	0.00	1.71	1.71
None of the above	n/a	n/a	1.71	1.71

*n/a* not available

#### Additional criteria

We asked survey respondents to list additional criteria they used (if any) when selecting implementation theories, outside of the 19 listed in the survey. After eliminating responses that overlapped conceptually with the 19 listed criteria and synthesizing conceptual duplicates, 3 additional criteria were identified (see Table 2): *familiarity* (extent to which principal investigator or research team is familiar with the theory), *degree of specificity* (extent to which included constructs are comprehensive of implementation determinants or specific to a particular set of implementation determinants), and *accessibility* (extent to which non-experts are able to understand, apply, and operationalize a theory’s proposition).

#### Qualitative responses

Some respondents expressed concern that political criteria sometimes held more weight than scientific criteria. For example, a UK-based, PhD-prepared implementation researcher noted, “In my experience, frameworks are often selected for the wrong reasons. That is, the basis for selection is political rather than scientific.” Indeed, one US-based, PhD-prepared implementation researcher suggested adding the criterion, “My advisor told me to!” Many respondents reported that the selection of implementation theories is often haphazard or driven by convenience or prior exposure. As a representative example, a US-based, PhD-prepared implementation researcher wrote, “To some degree selection is arbitrary. There are probably several theories that would be fruitful, and I tend to use ones that are familiar to me.” A US-based physician researcher wrote, “I wish there were a simple, systematic process for selecting theories!”

## Discussion

An international sample of implementation scientists collectively reported using more than 100 theories to inform implementation planning and evaluation to guide data collection and analysis, characterize features of the project environment and relationships between key constructs, and guide interpretation and dissemination of project outcomes. The theories that were most commonly used, including the CFIR, TDF, and Diffusion of Innovations, were used by respondents from nine different countries across four continents. Some respondents developed theories “in-house,” adapted existing theories, or combined components of multiple theories to meet the needs of their project.

Findings indicate that implementation scientists use a large number of criteria to select theories. It is possible that this large number reflects the varying sets of criteria that implementation scientists must consider depending on a theory’s intended use. (We describe this possibility in more detail below.) It may also be possible that the large number of criteria that implementation scientists consider when selecting theories reflects a lack of clarity regarding how to select theory. Indeed, our findings suggest that there is little consensus on which criteria are the most important. This may contribute to the persistent underuse, superficial use, and misuse of theories [4–8].

Our qualitative results suggest that the process for selecting implementation theories is often haphazard or driven by convenience or prior exposure. Selecting theories based on convenience or prior exposure may deepen knowledge about a given theory with repeated use; however, doing so also has the potential to limit theories’ benefits, particularly if theories are poorly suited to users’ objectives (e.g., selecting implementation strategies, framing study questions, motivating hypotheses). Convenient or familiar theories may contribute to silos in the field, limiting our ability to generalize findings, promote progress, and promote shared understanding.

This study had several limitations. We opted not to conduct a systematic literature review to develop the survey because the literature on this topic is likely diffuse and difficult to identify; thus, we began with key contributions of which we were aware. We included open-ended items to address the bias associated with this approach; however, we acknowledge that the criteria listed in the survey may have influenced responses. Though we tried to capture variation across respondent characteristics, our survey sample might not have been fully representative of perspectives in the field. For example, we did not have as many practitioner respondents or as many respondents from outside North America and Europe as we hoped; nevertheless, this appears to be the first attempt to assess the criteria that implementation scientists use to select theory, and future efforts to understand and streamline theory use should further consider the perspectives of these groups. Additionally, since we recruited a convenience sample, those who completed the survey may be systematically different from those who opted not to complete the survey. We did not ask respondents about the extent to which they value theory in their work. Indeed, we acknowledge that some implementation scientists do not view theory as critical to their work (e.g., [24, 25]).

## Conclusions

Our results suggest that implementation scientists may benefit from guidance for theory selection. Developing such guidance is challenging given potential variation in implementation scientists’ roles, priorities, and objectives, limiting the benefit of prescriptive guidance. Indeed, there may not be one “best” theory for a given project. Instead, implementation scientists will benefit from considering the broad range of criteria that we propose in this paper.

The field of implementation science will benefit from transparent reporting of the criteria that implementation scientists use to select theories. Transparent reporting may encourage implementation scientists to carefully consider the relevance of a selected theory instead of defaulting to theories that are convenient or familiar but are poorly suited to implementation scientists’ objectives. In turn, transparent reporting may diminish silos in the field by making explicit scientists’ thinking in selecting a particular theory, thus promoting progress through generalizable findings and shared understanding.

Examples of transparent reporting of the criteria that we identified in this study (or any others) exist. Birken et al. [26] justified the use of a taxonomy of top manager behavior [27] to explore of the relationship between top managers’ support and middle managers’ commitment to implementation by describing three categories of behavior in which top managers might engage to promote middle managers’ commitment (i.e., logical consistency, description of a change process). Alexander et al. [28] described using Klein and Sorra’s [29] theory of innovation implementation to assess the influence of implementation on the effectiveness of patient-centered medical homes [30] because the theory explains how the proficient and consistent use of an innovation influences its effectiveness (i.e., outcome of interest, specificity of causal relationships among construct). Yet transparent reporting of criteria used to justify theory selection is limited. Requiring manuscripts to include a section describing the criteria used to justify theory selection may promote more consistent reporting.

Several areas of future research would extend our initial attempt in this paper to explore criteria for selecting implementation theories. Specifically, the criteria that implementation scientists use to select theory may relate to how they intend to use theory. Understanding this relationship would help to refine the criteria presented here. We also recognize that substantive differences between theories and frameworks likely have implications for the criteria used to select them. For example, specificity of causal relationships among constructs is likely to be of greater importance for selecting theories than frameworks. We are currently refining the criteria and working to develop a useful, practical, and generalizable checklist of criteria based on concept mapping by implementation scientists [31]. The exercise will categorize the criteria and rate their clarity and importance. Our goal is for the checklist to take into account how and what kind of theory implementation scientists intend to use. This represents a first step toward what we hope will be a continued effort to refine the criteria, thus promoting more consistent and appropriate use of theory in implementation science and more effectively building the range of knowledge necessary to help ensure successful implementation across diverse settings.

## Abbreviations

CFIR: Consolidated Framework for Implementation Research
PARIHS: Promoting Action on Research Implementation in Health Services
RE-AIM: Reach Effectiveness Adoption Implementation Maintenance
SD: Standard deviation
TDF: Theoretical Domains Framework
